# SARS-CoV-2 diagnostic diary: from rumors to the first case. Early reports of molecular tests from the military research and diagnostic institute of Rio de Janeiro

**DOI:** 10.1590/0074-02760200200

**Published:** 2020-07-13

**Authors:** Marcio da Costa Cipitelli, Elizabeth Valentin, Nadia Vaez Gonçalves da Cruz, Tatiana LS Nogueira, Elaine Cristina Amaro de Melo, Rebeca Araujo da Silva, Marcelo M Serra, André L Meriano, Alberto ML Colares, Marcos Dornelas-Ribeiro, Caleb GM Santos

**Affiliations:** 1 Instituto de Biologia do Exército, Laboratório de Biologia Molecular, Rio de Janeiro, RJ, Brasil; 2 Instituto de Biologia do Exército, Laboratório de Biodefesa, Rio de Janeiro, RJ, Brasil; 3 Instituto de Biologia do Exército, Laboratório de Genética, Rio de Janeiro, RJ, Brasil

**Keywords:** coronavirus, SARS-CoV-2, RT-PCR, global pandemic, COVID-19

## Abstract

Corona virus disease (COVID-19) presents a serious threat to global health. A historical timeline of early molecular diagnostics from government alert (January 22) (D) was presented. After *in silico* analysis, Brazilian Army Institute of Biology (IBEx-RJ) tested samples *in house* using real-time reverse transcriptase polymerase chain reaction (RT-PCR) (fast mode) based on Centers for Disease Control and Prevention (CDC) recommendations. First cases from Brazil, Rio de Janeiro, IBEx, and diagnosis team were reported in D36, D44, D66, and D74 respectively. Therefore, after 1300 tests, we recommend N1/N2 primer sets (CDC) for preliminary and Charité protocol confirmation in case of positive results. Moreover, every professional should be tested before starting work, in addition to weekly tests for everyone involved.

According to the World Health Organization (WHO), viral diseases continue to emerge and present a serious threat to global health.^1^ ^,^ ^2^ ^,^ ^3^ Viral epidemics such as severe acute respiratory syndrome (SARS), Middle East respiratory syndrome (MERS), and the recent coronavirus disease (COVID-19), all caused by coronavirus subtypes, clearly illustrate the grave danger posed by these pathogens.^4^ ^,^ ^5^ ^,^ ^6^

The tools used for the accurate diagnosis of these viral infections must have high sensitivity, specificity and preferably, affordability for the benefit of the entire population.^7^ The diagnosis of pathologies related to respiratory viruses in Brazil is mostly clinical, with low requisition of confirmatory laboratory tests, which results in the underreporting of these infections.^8^ Polymerase chain reaction (PCR) single plex tests have emerged in recent times, but they possess low resolvability due to the similarity in clinical signs and symptoms shared by many pathogens that cause respiratory viral infections.^9^ Thus, these tests have gradually evolved into viral multiplex PCR panels with great prospects for diagnostic improvement. However, with the cost reaching up to $500 per patient, multiplex tests are expensive and inaccessible to the majority of population.^9^ In addition to pathogens such as rhinovirus, influenza, and H1N1, the limited panels currently available also screen for four subtypes of coronavirus already occurring in our environment: 229E, NL63, HKU1, and OC43.^10^

The Brazilian Armed Forces, represented by its health system and the Chemical, Biological, Radiological, and Nuclear defense system (CBRN), mobilise their resources to tackle the relevant threats that reach national/international levels and require actions of biological defense. In Brazil, in accordance with the federal law, ensuring effective biosafety is one of the responsibilities of the Brazilian Army. The Army’s role in this area makes it a key player in the public health services for military personnel in peacetime and keeps it trained for providing medical aid in times of conflict. A part of this system includes the Brazilian Army Institute of Biology (IBEx), a clinical research institute closely related with other Brazilian research institutions, such as Federal University of Rio de Janeiro (UFRJ) and Oswaldo Cruz Institute (IOC/Fiocruz). IBEx received clinical samples of military personnel (active or retired) and their dependents from Rio de Janeiro and São Paulo, the two most important cities in Brazil. The objective of this work is to present a historical timeline of pandemic dynamics in a reference laboratory and contribute to diagnostic methods in face of the growing need for tests.

The study was approved (30918520.4.0000.9433) by the Research Ethics Committee of Centro de Capacitação Física do Exército (CCFEx). Initially, an alert was issued to us on January 22 (D) about the imminent possibility of COVID-19 cases in Brazil. As a result, GenBank was searched for sequences pertaining to the viral agent. Surprisingly, five complete genomes were found, some fragments, and a reference sequence for the new coronavirus, severe acute respiratory syndrome coronavirus 2 (SARS-CoV-2) dated December 2019. The reference sequence was aligned (ClustalW) with the sequences of SARS, MERS, and four types of coronaviruses (229E, NL63, HKU1, and OC43), which were included in our multiplex PCR-based tests. From this, a phylogenetic tree (Fig. 1) was built using the maximum likelihood method based on the Tamura-Nei model in MEGA software.^1^

**Fig. 1:**
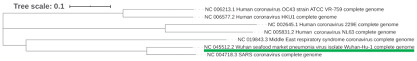
preliminary phylogenetic tree of simple reference genomes from important coronavirus subtypes. Highlighted, the sequence name referring to SARS-Cov-2 in January 22.

The new viral sequences were very different from all others, however, they were found to be closer to SARS.^2^ Moreover, based on the limited information available regarding the primers of commercial respiratory panels, we concluded that it would not be possible to detect the new SARS-CoV-2 using these panels. From January 29 (D8) onwards, we had access to the primer sequences released by Centers for Disease Control and Prevention (CDC), intended to be used for the purpose of the detection of the virus (Table I).

**TABLE I:** Primers suggested by Centers for Disease Control and Prevention (CDC) previously

Name	Sequence	Conc
2019-nCoV_N1 Forward Primer	5’-GAC CCC AAA ATC AGC GAA AT-3’	20 μM
2019-nCoV_N1 Reverse Primer	5’-TCT GGT TAC TGC CAG TTG AAT CTG-3’	20 μM
2019-nCoV_N1 Probe	5’-FAM-ACC CCG CAT TAC GTT TGG TGG ACC-BHQ1-3’	5 μM
2019-nCoV_N2 Forward Primer	5’-TTA CAA ACA TTG GCC GCA AA-3’	20 μM
2019-nCoV_N2 Reverse Primer	5’-GCG CGA CAT TCC GAA GAA-3’	20 μM
2019-nCoV_N2 Probe	5’-FAM-ACA ATT TGC CCC CAG CGC TTC AG-BHQ1-3’	5 μM
2019-nCoV_N3 Forward Primer	5’-GGG AGC CTT GAA TAC ACC AAA A-3’	20 μM
2019-nCoV_N3 Reverse Primer	5’-TGT AGC ACG ATT GCA GCA TTG-3’	20 μM
2019-nCoV_N3 Probe	5’-FAM-AYC ACA TTG GCA CCC GCA ATC CTG-BHQ1-3’	5 μM

The preliminary similarity analysis using Primer-BLAST showed that the disclosed primers could detect the SARS-CoV-2 genome, based on the sequences deposited in the NCBI. The specificity was predicted as ideal for the primer named N1. Thus, on February 20 (D30), in line with international standards, the primers and probes recommended by the CDC protocol were procured (“N” Gene). B-actin gene served as an internal control to human DNA. Biological samples were obtained using three synthetic oropharyngeal swabs (nose - 2, throat - 1); and RNAs were extracted mainly using QIAmp RNA Viral mini kit and QIAcube automated platform (Qiagen, Hilden, Germany).

Two strategies were followed for the diagnosis of SARS-CoV-2 positive cases. In the first strategy, patients with similar signs and symptoms were tested for the presence of other viral agents using the 21-multiplex respiratory viruses panel using real-time PCR (21VIR) (Mobius Life, Curitiba, PR) (Fig. 2). If they were positive for any of the viruses in this assay, considering the hypothesis of co-detection, we assumed that there would be a decreased chance of infection by SARS-CoV-2. The second strategy included the active search for SARS-CoV-2 using the PCR direct detection test, in accordance with the CDC protocol.

**Fig. 2:**
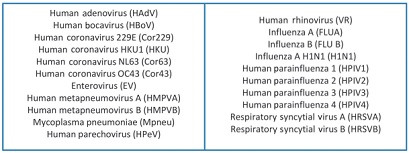
21VIR panel pathogens detectable in our laboratory.

The direct *in house* detection of SARS-CoV-2 followed the basic real-time reverse transcriptase (RT)-PCR protocol of the commercial kit GoScript Probe 1-step qPCR Master Mix (Promega, Madison, WI) on StepOnePlus (Applied Biosystems, Foster City, CA) and CFX96 (Bio-Rad, Hercules, CA) thermocyclers. Anticipating a possible increase in demand for tests, protocol was adapted to the “fast” format and the processing time was decreased to 40 min (Table II).

**TABLE II:** Cycle conditions for SARS-Cov-2 real-time reverse transcriptase polymerase chain reaction (RT-PCR) detection based in Centers for Disease Control and Prevention (CDC) primers

Steps	Cycles	Temperature	Time
Reverse transcription	1	45ºC	5 min
Reverse transcriptase inactivation and DNA Polymerase activation	1	95ºC	2 min
Denaturation	40	95ºC	3 sec
Annealing and extension	40	60ºC	30 sec

On February 26 (D36), the Brazilian Health Ministry confirmed first case of the new coronavirus in Brazil. Up to that date, suspicious cases from military related personnel had been referred to IBEx for evaluation, all of which turned out to be negative.

On March 5 (D44), the first case in the State of Rio de Janeiro was confirmed by the Brazilian government. On March 12 (D51), the first case of local transmission was confirmed in the city of Rio de Janeiro, with no history of travel to countries with community transmission. On March 12 (D51), a presumptively positive virus sample was obtained from a Reference Public Laboratory (RPLab) in a suitable viral transport media. Surprisingly, the sample tested negative for 21VIR and SARS-CoV-2 *in house* tests. On March 16 (D55), an alternative diagnostic kit, manufactured in Brazil (Bio-Mangui-nhos/Fiocruz) recommended by Ministry of Health and based on a second protocol from Charité, Berlim (E gene) was obtained together with new samples from the RPLab. From then on, IBEx began to provide diagnostic assistance for the civilian samples from RPLab. The virus sample obtained on March 12 was tested again and no virus was detected.

The test for SARS-CoV-2 tested negative samples from civilian and military personnel until March 17 (D56), when samples received from the RPLab were positive for the first time, as per the Charité protocol.^3^ They were also confirmed as positive by our protocol. Additional tests and literature reports showed that N2 primers were not specific for SARS-CoV-2 virus.^4^ Shortly afterwards, the CDC removed the sequences from its website and suggested commercial tests based on them. On the same day, the first confirmed death by COVID-19 in Brazil was reported.

After March 18 (D57), the diagnosis started to be based on the direct search for SARS-CoV-2, due to the low availability of 21VIR and the need to direct the lab workers to perform the COVID-19 tests. The 21VIR panels were used only for inpatients. Positive tests for COVID-19 were mandatorily confirmed by both the protocols before being released.

In the Fig. 3, the results of viruses detected from the 21VIR panel are summarised. Of the first 175 samples analysed (D30-D91), 118/2 were negative/inconclusive, and 55 were positive for 21VIR viruses. Among the viruses detected, a predominance of positive cases of Rhinovirus was found (~ 29%). But the great diversity of viruses circulating in Brazil showed that single plex traditional tests would not be effective for diagnosis.

**Fig. 3:**
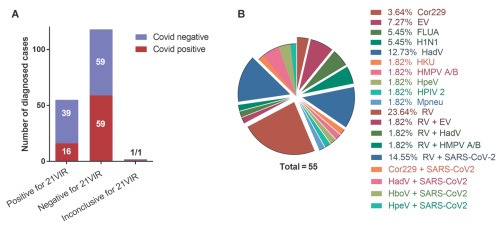
(A) diagnosed cases of 129 tests of 21VIR related to SARS-CoV-2 results. (B) Proportion of respiratory virus detected and codetected in our investigation. RV: rhinovirus: EV: enterovirus; FLUA: influenza A; H1N1: influenza A (swine); HadV: adenovirus; Cor229: coronavirus subtype 229; HKU: coronavirus subtype HKU1; HMPV A/B: metapneumovirus subtypes A or B; HPIV2: parainfluenza 2; Mpneu: Micoplasma pneumoniae (bacteria)*; HpeV: parecovirus; SARS-CoV2: coronavirus COVID-19.

On March 18 (D57), the first military related patient with COVID-19 were diagnosed. The results and timeline are summarised in Fig. 4. On March 27 (D66) the first positive case of a military officer from institute was reported. Subsequently, on the same day (D66) a military officer assigned to diagnostic team tested positive. On March 30 (D69), first rapid immunological kits arrived in Brazil.

**Fig. 4:**
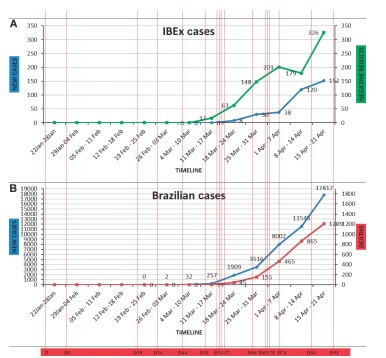
(A) timeline of new cases diagnosed and negative results by our Institute. (B) Timeline of new cases diagnosed and deaths obtained from Brazilian Health Ministery.

Until March 31 (D70), all results were similar between the two PCR-based diagnostic protocols. However, between April 1-14 (D71-D84), 28 tests were inconclusive to CDC protocol. So, the Charité protocol was particularly important to confirm the results. On March 31 (D70), upon receiving a request from the RPLab, we evaluated the new commercial 2019-nCoV RT-PCR diagnostic panel suggested by the CDC,^5^ using 14 previously positive samples for our *in house* CDC protocol and confirmed by the Charité protocol. All the results were found consistent.

Under these conditions, we suggest for now the use of a combination of N1/N3 or preferably N1/N2 primer sets (CDC protocol) for preliminary tests considering that the CDC removed N3 primer set from its recommendation on March 15 (D54), and mandatory confirmation of positive results using E gene from Charité protocol. n. Using this configuration, from around 1331 tests, it was found 348 (26,5%) positives, 934 (71,1%) and only 31 (2,4%) remain inconclusive. Negative results may be obtained from CDC N1/N2 primers sets corroborating recent findings about higher sensitivity of N1 primer set.^3^

On April 04 (D74), the first member of our molecular diagnostic team (MDT) tested positive for COVID-19. Until April 14 (D84), nine members of institute (~ 4%) tested positive (two of our team). The first two positives from D66, retested one negative and the other remain positive without any symptoms, 18 days after diagnostic. Finally, on April 21 (D91) the last day of report, 27 Institute members (~ 10%) tested positive and were away from work.

An important aspect of COVID-19 is the fact that symptoms appear only a few days after close contact with an infected person or contaminated surfaces. Hence, the daily statistics of new cases represent people who may have had contact a few days before a positive diagnosis. Presymptomatic transmission in such cases is a possibility, and all of this should be considered while making epidemiological decisions.

Curiously, including 21VIR we found around 20% of codetection including rhinovirus (RV), around 15% of co-detection of RV/SARS-CoV-2 and a higher SARS-CoV-2 positivity in negative patients for 21VIR. Therefore the positive cases for 21VIR do not exclude the possibility of co-detection or co-infection by SARS-CoV-2.^5^ These panels are a promising tool for understanding the cycles, seasonality, and multitude of these viral respiratory pathologies, which are a major cause of clinical visits worldwide.^6^ The discrepancies observed between the two SARS-CoV-2 PCR protocols as well as the negative result of the first sample obtained from the RPLab may be due to some degree of degradation of the RNAs received, sensitivity levels discrepancies or unspecific amplifications recently described. But it is possible that multiple strains are circulating.^7^ Subsequent sequencing data analysis will uncover this possibility. So, the molecular biology plasticity strategies related to genomes underline the importance of involving professionals with the relevant expertise in providing technical, scientific, and diagnostic support to clinical analysis.

The data referring to confirmed cases have a bias regarding the time required for adaptation, planning, logistics, and definition of diagnostic protocols in national laboratories. Moreover, the objective of this brief report is to verify the molecular techniques and can change considerably in view of the distribution of other rapid diagnostic kits.^8^ The first case in this institute was on D66. The MDT technicians are experienced in the use of personal protective equipment (PPE) and in biological defense protocols. The temporal dynamics may be different in diagnostic laboratories with other characteristics.

With the increase in cases,^9^ new professionals will need to be recruited for the collection, diagnostic and medical care teams. However, it is suggested that every professional be tested before starting work, in addition to weekly tests for everyone involved. It should be noted that about 20% of responding health-care workers were infected in Italy.^10^ In addition to individual risk, the withdrawal of professionals greatly reduces the ability to respond to the pandemic. New strategies for redeployment of personnel who have recovered from COVID-19 may be planned. Instead of the possibility of reinfection stays unclear, preliminary some reports suggest no recurrence after re-exposure of COVID-19 in non-human primate models.^11^

For the future, the widespread utilisation of new sequencing technologies, user-friendly bioinformatics tools and the appropriate use of sequencing tools in clinical diagnosis will aid in the development of targeted therapy. Sequencing approaches based on targeted amplicons or metagenomics may be powerful tool in detecting the real pathogens in every sample, coinfections, multiple strains, phylogenomical, and phylogeographical interpretations and will contribute to faster and more accurate responses in epidemics
